# Characteristics, management and outcomes of critically ill patients who are 80 years and older: a retrospective comparative cohort study

**DOI:** 10.1186/1471-2253-14-126

**Published:** 2014-12-20

**Authors:** Hasan M Al-Dorzi, Hani M Tamim, Shihab Mundekkadan, Muhammad R Sohail, Yaseen M Arabi

**Affiliations:** College of Medicine, King Saud bin Abdulaziz University for Health Sciences and Intensive Care Department, King Abdulaziz Medical City, Riyadh, 11426 Kingdom of Saudi Arabia; King Abdullah International Medical Research Center, Riyadh, 11426 Kingdom of Saudi Arabia; Department of Internal Medicine, American University of Beirut-Medical Center, Beirut, Lebanon; Intensive Care Department, King Abdulaziz Medical City, Riyadh, 11426 Kingdom of Saudi Arabia; Intensive Care Department, King Abdulaziz Medical City, Riyadh, Saudi Arabia; College of Medicine, King Saud bin Abdulaziz University for Health Sciences, PO Box 22490, Mail code 1425, Riyadh, 11426 Kingdom of Saudi Arabia

**Keywords:** Aged 80 and over, Critical illness, Health outcome, Mortality

## Abstract

**Background:**

Older age is associated with chronic illnesses and disability, which contribute to increased admission to the intensive care unit (ICU). Our primary objective was to compare the characteristics, ICU management and outcomes of critically ill patients ≥ 80 year-old with those of younger patients.

**Methods:**

This was a retrospective cohort study of patients admitted to a tertiary-care ICU from 1999 to 2011. The characteristics, ICU management and outcomes of patients ≥ 80 year-old were compared with those 50–64.9 and 65–79.9 year-old. Multivariate analysis was performed to determine the adjusted risk of Do-Not-Resuscitate orders and hospital mortality in patients ≥ 80 year-old compared with the younger groups.

**Results:**

During the study period, patients aged ≥ 80 years (N = 748) represented 7.9% of all ICU admissions and 12.8% of patients aged ≥ 50 years. Chronic cardiac (32.2%) and respiratory (21.8%) diseases were more prevalent in them than the younger groups (p < 0.0001). The most common reasons for their ICU admission were cardiovascular (30.9%) and respiratory (40.4%) conditions. Sepsis was commonly present in them on admission (32.9%). Mechanical ventilation and renal replacement therapy were commonly provided (76.9% and 16.0%, respectively). During ICU stay, Do-Not-Resuscitate orders were more frequently written for patients aged ≥ 80 years (35.0%) compared with 21.9% for 50–64.9 year-old group, p < 0.0001, and 25.4% for the 60–79.9 year-old group, p < 0.0001. On multivariate analysis, patients aged ≥ 80 years were more likely to receive these order compared with the 50–64.9 year-old patients (adjusted OR, 1.83; 95% CI, 1.45-2.31) and the 65–80 year-old patients (adjusted OR, 1.64; 95% CI, 1.32-2.04). The hospital mortality increased gradually with age and was highest (54.6%) in patients ≥ 80 year-old (p < 0.0001). Patients ≥ 80 year-old had higher risk of hospital mortality compared with patients aged 50–64.9 years (adjusted OR, 2.16; 95% CI, 1.73-2.69) and with those aged 65–79.9 years (adjusted OR, 1.51; 95% CI, 1.23-1.86).

**Conclusions:**

Patients ≥ 80 year-old represented a significant proportion of ICU admissions. Although they received life sustaining measures similar to younger groups, they had higher adjusted mortality risk compared with the younger groups.

## Background

Old age is associated with chronic medical diseases and functional impairment, which may lead to increased incidence and severity of acute critical illnesses and to admission to the intensive care unit (ICU). For severe sepsis, the incidence generally increases with age during adulthood [1]. Incidence of hospitalization secondary to community-acquired pneumonia doubles in patients aged > 60 years [2]. A study from the United Kingdom found that the ratio of ICU admissions to local population in people ≥ 60 years rose linearly by 2.62 admissions per 10,000 population per year over a six consecutive year-period with the increase being highest in patients ≥ 80 years [3].

Rationing health care based upon age has been reported. In a systematic review, Sinuf et al. studied rationing of ICU resources and found that age and severity of illness were most strongly associated with a refusal to admit to the ICU [4]. A recent prospective cohort study of patients > 85 year-old presenting to the emergency departments of 15 Parisian hospitals found significant variability in ICU admission even after adjustment for patient characteristics [4]. This was likely related to the belief that older age was associated with poor outcomes after ICU admission [5]. This issue has been investigated in multiple studies mostly from Western countries [6–8]. However, it is also believed that age explains only a small part of the outcomes of critical illness and that prior functional status, co-morbidities and the level of therapeutic support are important factors [9].

Knowing the outcomes and prognosis determinants of patients aged ≥ 80 years who are admitted to the ICU is important for clarification of perceptions of intensive care providers and possibly for proper allocation of resources. The objectives of this study were to describe the characteristics, management and outcomes of critically ill patients ≥ 80 year-old and to determine if age ≥ 80 years was an independent predictor of ICU management and of hospital mortality in a tertiary-care center in Saudi Arabia.

## Methods

### Patients and setting

This was a retrospective analysis of a cohort of adult patients admitted between January 1, 1999 and December 31, 2011 to the ICU of King Abdulaziz Medical City, a 900-bed tertiary-care teaching hospital in Riyadh, Saudi Arabia that had been accredited by the Joint Commission International. The ICU was a 21-bed medical-surgical closed unit and was staffed by board-certified critical care physicians on a 24 hours per days, 7 days a week basis [10] with residents from different specialties rotating periodically. In this study, we compared patients ≥ 80 year-old with those 50–64.9 and 65–79.9 year-old because the younger patients (< 50 year-old) were thought to have completely different comorbid conditions, reasons for ICU admission and end-of-life care. For patients who had multiple ICU admissions in the same hospitalization, we included only the first ICU admission. The Institutional Review Board of King Saud bin Abdulaziz University for Health Sciences, Riyadh, Saudi Arabia approved this study, granted waiver of consent and allowed the review of the patients’ medical records.

### Collected data

Our Intensive Care Department had a comprehensive database in which trained coordinators prospectively collected demographic and clinical data and followed patients for predefined outcomes. The following data were extracted from the database: age, gender, body mass index, functional status before hospitalization based on the modified Rankin Scale [11], Acute Physiology and Chronic Health Evaluation (APACHE) II score [12], the presence of chronic health illnesses as defined by APACHE II system, the main reason for ICU admission (as per APACHE II definitions), presence of sepsis on admission, diagnosis of the following on ICU admission: myocardial ischemia such as acute coronary syndrome and acute myocardial infarction, community- or hospital-acquired pneumonia and new stroke, admission Glasgow Coma Scale score and admission platelet count, creatinine, lactate, bilirubin and International Normalized Ratio (INR).

We also noted the following ICU management aspects: use of vasopressors within the first 24 hours after ICU admission, requirement for mechanical ventilation, renal replacement therapy and tracheostomy during ICU stay, performance of cardiopulmonary resuscitation for cardiac arrest while in the ICU and the practice of Do-Not-Resuscitate orders. During the study period, advanced directives were not practiced in Saudi Arabia. However, our hospital had a policy in which a Do-Not-Resuscitate order can be written if three qualified physicians agreed that a patient would not benefit from cardiopulmonary resuscitation in case of cardiac arrest because of he/she was terminally ill or had a severe illness with high predicted mortality, especially if significant functional disability and/or dementia preexisted. This medical decision would be then explained to the patient or surrogate decision maker who generally had to agree before its implementation. His or her signature was not required. Do-Not-Resuscitate orders generally precluded intubation but not the use of noninvasive ventilation.

In this study, hospital mortality was the primary outcome. Other outcomes included ICU and post-ICU discharge mortality, length of stay in the ICU and hospital and duration of mechanical ventilation. We also calculated the predicted mortality based on the Mortality Probability Model (MPM) II at 0 and 24 hours [13] and on APACHE II score for the three age groups.

### Statistical analysis

Statistical analysis was done using Statistical Analysis System (SAS, version 9.0; SAS Institute, Cary, NC). Continuous variables were reported as means with standard deviations and if clinically relevant as medians with the first and third quartiles. Categorical variables were presented as frequencies with percentages. The standardized mortality ratio for APACHE II was calculated for each group by dividing the actual by predicted hospital mortality and reported with its 95% confidence intervals (CI) [14]. The Chi-squared test was used to assess the differences between categorical variables and the Student’s t-test to assess the differences between continuous variables. Multivariate analysis was performed to evaluate older patients’ adjusted risk of hospital mortality, having a Do-Not-Resuscitate order during ICU stay and need for renal replacement therapy. The association between hospital mortality and age was also evaluated in patients admitted to the ICU because of cardiovascular, respiratory, neurological, other medical, trauma-related and postoperative reasons and in patients with the diagnosis of myocardial ischemia, community- or hospital-acquired pneumonia and new stroke on ICU admission. Multivariate analysis was also performed to determine the predictors of hospital mortality in patients ≥ 80 year-old. The independent variables entered in these analyses were gender, nonage-related APACHE II score, the main reason for admission, chronic illnesses, functional status before hospitalization (moderately severe or severe disability versus more active status), mechanical ventilation, length of ICU stay, creatinine and INR. The results were presented as adjusted odds ratio (aOR) with 95% CIs. A P-value < 0.05 was considered statistically significant in all analyses.

## Results

### Characteristics of patients

**Figure 1:**
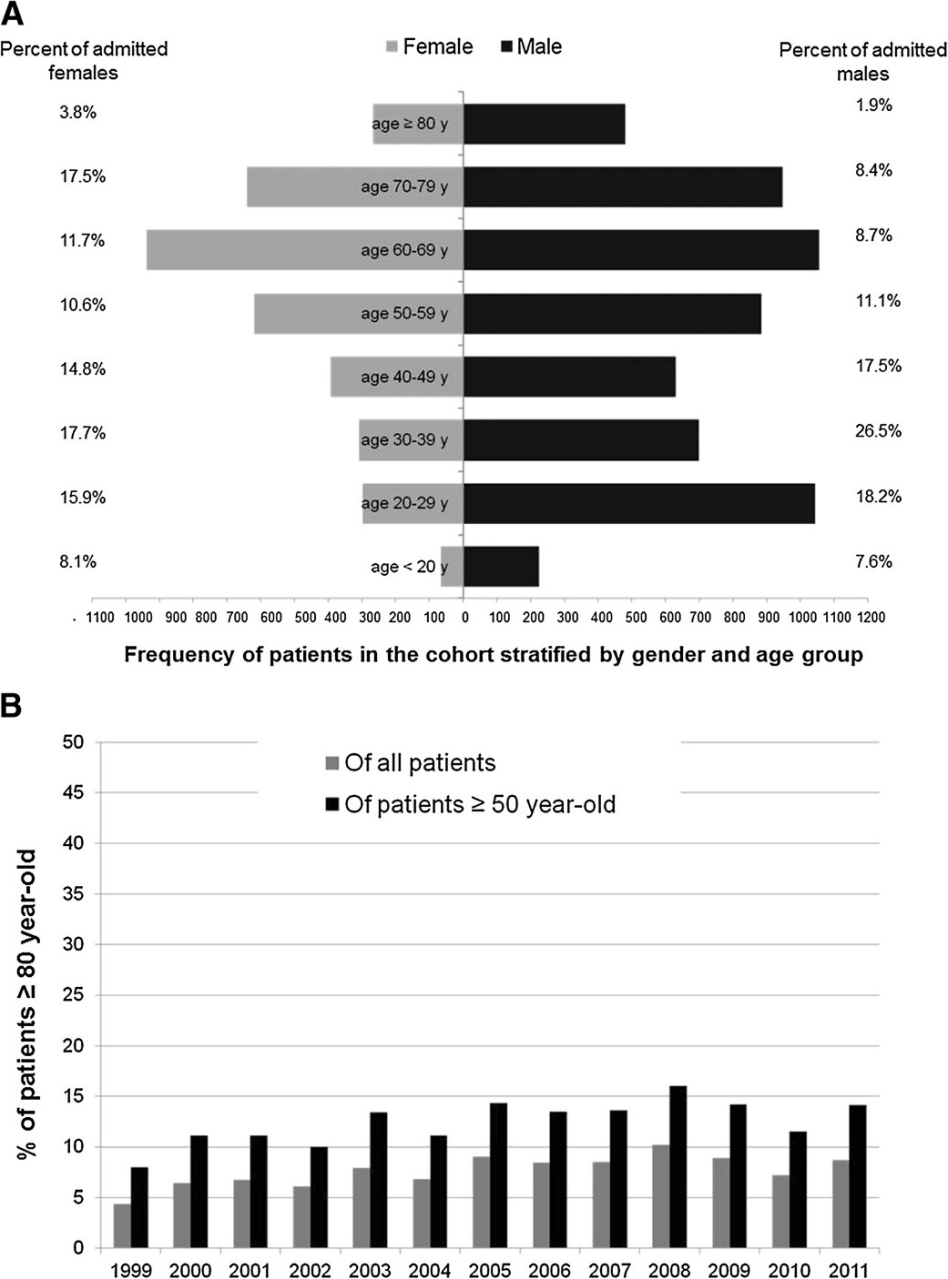
**Cohort description.** Panel **A**: Age distribution by decades of the cohort according to gender. Panel **B**: Percentages of patients aged ≥ 80 years old of all patients and of patients ≥ 50 years old admitted to the intensive care unit per admission year.

During the 13 year study period, 9493 patients were admitted to the ICU. The patients aged ≥ 80 years (N = 748) represented 7.9% of them and 12.8% of the 5832 patients who were 50 years and older. Their mean age was 85.1 ± 4.9 years (Q1-Q3: 81–88 years). Figure 1A describes the age distribution of the cohort according to gender. Men aged ≥ 80 years accounted for 8.1% of admitted men and women aged ≥ 80 years 7.6% of admitted women. Figure 1B describes the percentages of patients aged ≥ 80 years admitted from 1999 to 2011 and shows random variation between years. Additionally, Table 1 describes the characteristic of the three age groups. The oldest (≥80 year-old) patients were predominantly males (64.2%) with APACHE II score = 27.2 ± 8.2. Chronic cardiac (32.2%) and respiratory (21.8%) diseases were more prevalent in them than in the younger groups (p < 0.0001). More patients aged ≥ 80 years had moderate or severe physical disability before hospitalization as assessed by the modified Rankin Scale. Disorders of the respiratory (40.4%) and cardiovascular (30.9%) systems were their most common reasons for admission to the ICU. Sepsis was present on admission in 32.9%, the highest among the groups (p < 0.0001). Myocardial ischemia, community-acquired pneumonia and new stroke on admission were also more prevalent.

**Table 1:** **Patient characteristics**

	50-64.9 yr-old N = 2467	65-79.9 yr-old N = 2617	≥ 80 yr-old N = 748	P-value*	P-value**	P-value***
Age (years), mean ± SD	57.6 ± 4.3	71.1 ± 4.1	85.1 ± 4.9	<0.0001	<0.0001	<0.0001
Male gender, N (%)	1377 (55.8)	1507 (57.6)	480 (64.2)	0.20	<0.0001	0.0012
APACHE II, mean ± SD	23.3 ± 9.1	26.5 ± 8.7	27.1 ± 8.0	<0.0001	<0.0001	0.11
Non-age APACHE II, mean ± SD	20.5 ± 9.0	21.2 ± 8.7	21.1 ± 8.0	0.02	0.14	0.75
Body mass index (Kg/m^2^), mean ± SD	29.1 ± 7.7	28.4 ± 8.0	27.1 ± 7.9	0.02	<0.0001	0.001
Functional status^‡^ before admission, N (%)						
No significant disability	801 (32.6)	395 (15.3)	61 (8.2)	<0.0001	<0.0001	<0.0001
Slight disability	573 (23.4)	506 (19.5)	73 (9.8)	0.0006	<0.0001	<0.0001
Moderate disability	429 (17.6)	691 (26.6)	691 (20.0)	<0.0001	0.13	0.0003
Moderately severe disability	172 (7.0)	467 (18.0)	262 (35.2)	<0.0001	<0.0001	<0.0001
Severe disability	91 (3.7)	233 (9.0)	117 (15.7)	<0.0001	<0.0001	<0.0001
Chronic illnesses, N (%)						
Cardiac	413 (16.9)	644 (24.8)	240 (32.2)	<0.0001	<0.0001	<0.0001
Respiratory	328 (13.4)	475 (18.4)	162 (21.8)	<0.0001	<0.0001	0.04
Renal	379 (15.5)	476 (18.4)	120 (16.2)	0.007	0.68	0.16
Hepatic	511 (20.9)	354 (13.7)	46 (6.2)	<0.0001	<0.0001	<0.0001
Immunocompromised	349 (14.3)	303 (11.7)	56 (7.5)	0.007	<0.0001	0.001
Main reason for ICU admission, N (%)						
Cardiovascular	452 (18.3)	655 (25)	231 (30.9)	<0.0001	<0.0001	0.0001
Respiratory	862 (34.9)	1016 (38.8)	302 (40.4)	<0.0001	<0.0001	0.0001
Neurological	175 (7.1)	181 (6.9)	60 (8.0)	<0.0001	<0.0001	0.0001
Other medical	198 (8.0)	159 (6)	34 (4.6)	<0.0001	<0.0001	0.0001
Non-operative trauma	84 (3.4)	47 (1.8)	7 (0.9)	<0.0001	<0.0001	0.0001
Post operative	696 (28.2)	559 (21.4)	114 (15.3)	<0.0001	<0.0001	0.0001
Sepsis on admission, N (%)	723 (29.3)	723 (29.3)	838 (32)	0.04	0.06	0.65
Myocardial ischemia on admission, N (%)	227 (9.2)	367 (14.0)	151 (20.2)	<0.0001	<0.0001	<0.0001
Community-acquired pneumonia on admission, N (%)	207 (8.4)	348 (13.3)	99 (13.2)	<0.0001	<0.0001	0.96
Hospital-acquired pneumonia on admission, N (%)	83 (3.4)	95 (3.6)	33 (4.4)	0.61	0.18	0.32
New stroke on admission, N (%)	227 (9.2)	478 (18.2)	175 (23.4)	<0.0001	<0.0001	0.002
Admission GCS score, mean ± SD	10.5 ± 4.5	10.0 ± 4.5	9.2 ± 4.3	0.0002	<0.0001	<0.0001
Platelet count/μL, mean ± SD	212 ± 153	227 ± 153	243 ± 131	0.004	<0.0001	0.015
Lactate^¶^ (mmol/L), mean ± SD	3.8 ± 4.0	3.7 ± 4.0	3.5 ± 3.8	0.66	0.12	0.20
Creatinine^¶^ (μmol/L), mean ± SD	183 ± 175	190 ± 161	169 ± 130	0.18	0.052	0.005
Bilirubin* (μmol/L), mean ± SD	72 ± 138	44 ± 90	27 ± 61	<0.0001	<0.0001	0.0001
INR, mean ± SD	1.7 ± 1.1	1.7 ± 1.0	1.7 ± 1.1	0.66	0.6	0.88

APACHE, Acute Physiology and Chronic Health Evaluation; ICU, intensive care unit; INR, International Normalized Ratio; SD, standard deviation;

*P-value between patients aged 50–64.9 and those 65–79.9 years.

** P-value between patients aged 50–64.9 and those ≥ 80 years.

***P-value between patients aged 65–79.9 and those ≥ 80 years.

‡based on the modified Rankin Scale.

¶To convert creatinine to mg/dL divide by 88.4, bilirubin to mg/dL divide by 17.1, lactate to mg/dL divide by 0.111.

### Intensive care unit management

Table 2 describes certain aspects of ICU management for the three age groups. Use of vasopressors was similar in patients aged ≥ 80 years compared with the two younger groups. Patients aged ≥ 80 years had the highest rate of mechanical ventilation (76.9%) and the lowest rate of renal replacement therapy (16.0%). Multivariate analysis showed that the OR for providing renal replacement therapy was not different in patients aged > 80 years compared to 50–65 year-old patients (aOR, 0.824; 95% CI, 0.613-1.107) and 60–79.9 year old patients (aOR, 0.87; 95% CI, 0.65-1.15). Tracheostomy was performed more frequently in patients aged ≥ 80 years even though there was no difference in the duration of mechanical ventilation among the three groups.

**Table 2:** **Intensive care unit management**

	50-64.9 yr-old N = 2467	65-79.9 yr-old N = 2617	≥ 80 yr-old N = 748	P-value*	P-value**	P-value***
Use of vasopressors in the first ICU day, N (%)	975 (40.2)	1121 (43.6)	317 (43.0)	0.01	0.17	0.74
Requirement of mechanical ventilation, N (%)	1735 (70.4)	1863 (71.2)	575 (76.9)	0.50	0.0005	0.002
Renal replacement therapy, N (%)	536 (21.7)	554 (21.2)	120 (16.0)	0.63	0.0007	0.002
Tracheostomy, N (%)	318 (12.9)	387 (14.8)	142 (19.0)	0.05	<0.0001	0.005
Cardiopulmonary resuscitation in the ICU, N (%)	150 (6.1)	195 (7.4)	72 (9.6)	0.052	0.0008	0.052
Do-Not-Resuscitate order in the ICU, N (%)	542 (21.9)	663 (25.4)	262 (35.0)	0.005	<0.0001	<0.0001

ICU, intensive care unit.

*P-value between patients aged 50–64.9 and those 65–79.9 years.

** P-value between patients aged 50–64.9 and those ≥ 80 years.

***P-value between patients aged 65–79.9 and those ≥ 80 years.

**Figure 2:**
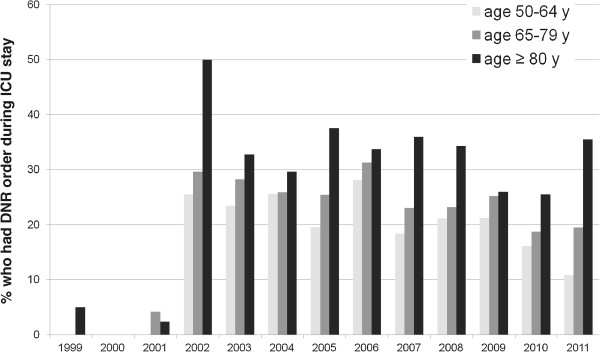
**Percentage of patients in the three age groups (50–64.9, 65–79.9 and ≥ 80 year-old) who had Do-Not-Resuscitate order during the stay in the intensive care unit per admission year.**

Figure 2 describes the percentages of patients in the different age groups who had Do-Not-Resuscitate orders during their ICU stay. Do-Not-Resuscitate orders were more frequently written for patients aged ≥ 80 years (35.0%) compared with 21.9% for 50–64.9 year-old group, p < 0.0001, and 25.4% for the 60–79.9 year-old group, p < 0.0001 (Table 2). In addition, Do-Not-Resuscitate orders were written earlier (median day after ICU admission, 3; Q1-Q3: 1–8 days) than for patients < 80 year-old (median, 5; Q1-Q3: 1–10 days for the 50–64.9 year-old and median, 5; Q1-Q3, 1–11 days for the 65–79.9 year-old). After controlling for other factors and compared with patients 50–64.9 years, the practice of Do-Not-Resuscitate orders was similar in patients who were 65–80 year-old (aOR, 1.16; 95% CI, 0.98-1.37) but higher for patients aged ≥ 80 years (aOR, 1.83; 95% CI, 1.45-2.31). Additionally, patients aged ≥ 80 years were more likely to have Do-Not-Resuscitate order compared with patients who were 65–80 year-old (aOR, 1.64; 95% CI, 1.32-2.04).

### Outcomes

Table 3 describes the outcomes of the patient groups. The lengths of stay in the ICU and hospital were similar in the three groups. Although there was no difference in ICU mortality between the three age groups, hospital mortality increased gradually with age and was highest (54.6%) in patients ≥ 80 years old (p < 0.0001). Moreover, the hospital mortality rates for patients who had Do-Not-Resuscitate order while in the ICU were 92.6% for the younger group, 89.7% for patients aged 65–79.9 years and 85.1% for the very elderly. The mortality rates of patients who had cardiopulmonary resuscitation for cardiac arrest while in the ICU were 82.7% for the younger group, 79.5% for patients aged 65–79.9 years and 80.6% for the very elderly.

**Figure 3:**
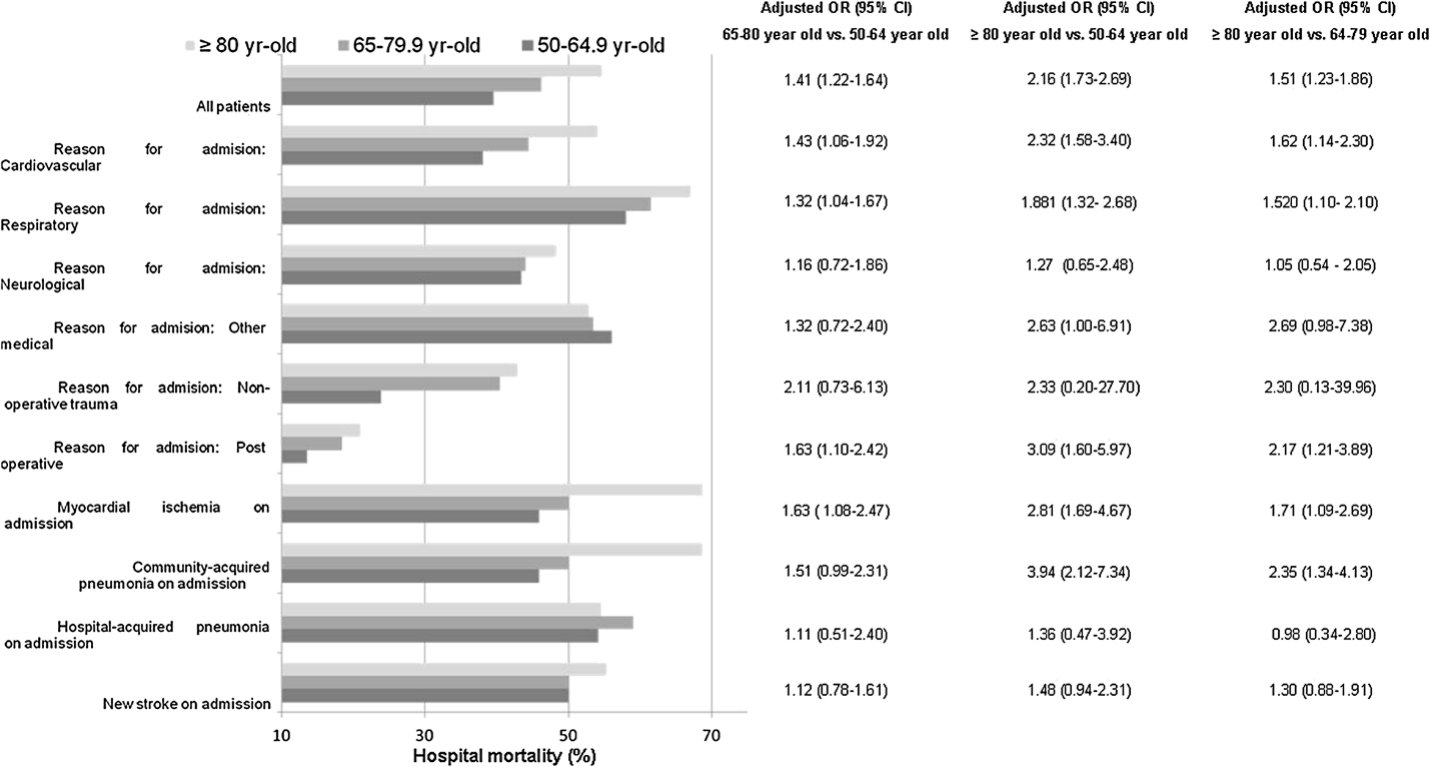
**Mortality rates of patients aged 50–64.9, 65–79.9 and ≥ 80 years stratified by various reasons for admission to the intensive care unit.**

Figure 3 describes the hospital mortality of the three age groups stratified by the reason of admission and generally shows that the mortality rates increased with age. This was not observed in patients admitted to the ICU because of hospital acquired pneumonia and neurologic disease. After adjusting for imbalances in the baseline characteristics, patients ≥ 80 year-old had higher risk of hospital mortality compared to patients aged 50–64.9 years (aOR, 2.16; 95% CI, 1.73-2.69) and those aged 65–79.9 (aOR, 1.51; 95% CI, 1.23-1.86). This increased risk was not observed in patients with ICU admission because of neurologic disorders, new stroke or hospital-acquired pneumonia (Figure 3).

**Table 3:** **Outcomes of patients in the three age groups**

	50-64.9 yr-old N = 2467	65-79.9 yr-old N = 2617	≥ 80 yr-old N = 748	P-value*	P-value**	P-value***
Duration of mechanical ventilation (days), mean ± SD	6.4 ± 10.5	6.4 ± 10.4	6.9 ± 8.8	0.97	0.85	0.87
ICU LOS (days), mean ± SD	7.8 ± 11.4	7.8 ± 11.1	7.4 ± 8.7	0.98	0.42	0.33
Hospital LOS (days), mean ± SD	44.2 ± 97.6	45.1 ± 136.8	48.7 ± 189.1	0.78	0.54	0.63
ICU mortality, N (%)	637 (25.8)	723 (27.6)	217 (29.0)	0.15	0.08	0.46
Post-ICU mortality of all ICU survivors, N (%)	340 (18.6)	486 (25.7)	193 (36.4)	<0.0001	<0.0001	<0.0001
Post ICU mortality of patients without Do-Not-Resuscitate orders, N (%)	270 (15.7)	386 (22.4)	129 (30.1)	<0.0001	<0.0001	0.0007
Post ICU mortality of patients with Do-Not-Resuscitate orders, N (%)	70 (63.6)	100 (59.5)	64 (62.1)	0.49	0.82	0.67
28-day mortality^#^, N (%)	719 (29.1)	838 (32.0)	275 (36.8)	0.03	<0.0001	0.02
Hospital mortality, N (%)	977 (39.6)	1208 (46.2)	410 (54.8)	<0.0001	<0.0001	<0.0001
Predicted mortality, mean % ± SD						
APACHE II	44.9 ± 28.0	53.8 ± 27.0	56.7 ± 25.7	<0.0001	<0.0001	0.02
MPM_0_ II	38.6 ± 30.1	46.7 ± 30.9	55.8 ± 29.0	<0.0001	<0.0001	<0.0001
MPM_24_ II	41.2 ± 31.3	48.4 ± 31.5	54.4 ± 30.0	<0.0001	<0.0001	<0.0001
Standardized mortality rate, (95% confidence interval)	0.88 (0.84-0.92)	0.86 (0.82-0.89)	0.97 (0.90-1.03)			

APACHE, Acute Physiology and Chronic Health Evaluation; ICU, intensive care unit; LOS, length of stay; MPM, Mortality Probability Model; SD, standard deviation.

*P-value between patients aged 50–64.9 and those 65–79.9 years.

**P-value between patients aged 50–64.9 and those ≥ 80 years.

***P-value between patients aged 65–79.9 and those ≥ 80 years.

# Patients discharged alive < 28 days after hospital admission were considered to be survivors.

Predictors of hospital mortality in patients who were 80 years or older were invasive mechanical ventilation (OR, 3.10; 95% CI, 1.96-4.90), chronic renal disease (OR, 1.75; 95% CI, 1.05-2.90), non-age APACHE II score (OR, 1.07; 95% CI, 1.04-1.10) and length of ICU stay (OR, 1.04 per one day increment; 95% CI, 1.02-1.07).

## Discussion

The main findings of this study were the following: patients aged ≥ 80 years represented a significant portion of patients admitted to the ICU; had significant comorbid conditions; were admitted mainly because of acute dysfunction of the cardiac and respiratory systems; and compared with the younger groups, received similar life sustaining treatments but were more likely to have Do-Not-Resuscitate orders and to die in the hospital than the younger groups.

The age structure of the world population has shifted and will continue to do so with the proportion of old people increasing in both developed and developing countries [15]. In parallel with this shift, more old patients are admitted to ICU. In Australia and New Zealand, the proportion of patients aged > 80 years was 13% of the adult ICU population and increased by 5.6% between 2000 and 2005 [16]. In Denmark, a study of 49,938 ICU admissions found that the proportion of patients aged ≥ 80 years increased from 11.7% of all ICU patients in 2005 to 13.8% in 2011 [17]. In the current study, patients aged ≥ 80 years constituted 7.9% of all patients admitted to the ICU between 1999 and 2011 and 12.8% of patients ≥ 50 year-old, with the proportion of admissions generally showing random variation from year to year. These findings are different than other studies [16, 17] likely because the Saudi population is much younger than those of developed countries. In 2011, people aged ≥ 80 years represented 0.6% of the population compared with 3.8% in Australia, 4.2% in Denmark and 3.8% in the United States [18].

Physicians frequently consider old age when deciding on the provision of life-sustaining measures. An observational simulation study found that 86, 78 and 62% of participating physicians (predominantly males without religious beliefs; median ICU experience = 9 years) felt that noninvasive mechanical ventilation, invasive mechanical ventilation or renal replacement therapy was warranted, respectively for patients aged ≥ 80 years [19]. On multivariate analysis, age < 85 years, self-sufficiency and bed availability were associated with ICU admission [19]. In a systematic review of 10 observational studies of seriously ill patients considered for ICU admission during periods of reduced bed availability, Sinuff et al. found that age and severity of illness were most strongly associated with a refusal to admit to the ICU [4]. A recent prospective study of patients > 85 years presenting to the emergency departments of 15 Parisian hospitals found significant variability in ICU admission even after adjustment for patients’ characteristics [5]. The geographic variation in ICU use for patients ≥ 85 years old was also seen in another study where it was less common in England (1.3%) than the United States (11.0%) [20]. In the current study, we have observed that patients aged ≥ 80 years were frequently provided with life support measures, such as mechanical ventilation and renal replacement therapy, like younger patients. However, age ≥ 80 years was found to be an independent risk factor for the practice of Do-Not-Resuscitate orders after controlling for co-morbid conditions.

Low physiological reserve and comorbidities often place very old people in a situation of greater complexity, which may impact outcome. However, it is thought that they have lived to that age because they are resilient to acute illnesses. Studies generally show higher critical illness-associated mortality in the old and very old patients. A study that classified patients into 75–79, 80–84 and ≥ 85 year age groups found that age was not associated with ICU mortality, but with long-term mortality (aOR: 2.17, for patients ≥ 85 years old and 1.82, for patients 80–84 years old) [6]. Another study found that patients aged 75–84 and ≥ 85 year-old had aORs of 1.38 (95% CI, 1.19-1.59) and 1.53 (95% CI, 1.29-1.81), respectively for 28-day mortality as compared with the 65–74 year-age group [7]. A secondary analysis of data from a randomized trial comparing the effects of dopamine and norepinephrine in patients with shock found that the mortality rates were higher in the old (75–84 years) and very old (≥ 85 years) patients at 28 days, at hospital discharge, and after 6 and 12 months [21]. Most very old patients were dead at 6 (92%) and 12 months (97%) with mortality rates increasing with age in all types of shock [21]. A retrospective Norwegian cohort study (n = 27,921) found that the hospital mortality was 21.4% in patients aged 50–79.9 years and 32.4% in patients aged > 80 years, who also received less mechanical ventilation (40.6% versus 56.1%) and had shorter median ventilatory support time (0.8 days versus 1.9 days) [22]. The mortality of the very elderly patients may be affected by admission type. A retrospective cohort study that the 30-day mortality of elderly patients (≥ 80 years) was 43.7% in medical, 39.6% in acute surgical, and 11.6% in elective surgical ICU patients with a corresponding adjusted 30-day mortality rate ratios compared with the 50–64 year-old patients were 2.7 (95% CI, 2.5-3.0) in medical, 2.7 (95% CI, 2.4-3.0) in acute surgical and 5.2 (95% CI, 4.1-6.6) in elective surgical ICU patients [17]. The adjusted mortality rate ratios for 31-365-day mortality among elderly patients were 2.5 (95% CI, 2.1-2.9) for medical, 2.2 (95% CI 1.9-2.5) for acute surgical and 1.9 (95% CI, 1.6-2.3) for elective surgical ICU patients [17]. A study that used Project IMPACT data for 124,885 patients treated from 2001 to 2004 found that mortality rates approximately doubled in the elective surgical group among patients aged in their 70s (2.4%), 80s (4.3%), and 90s (9.2%) but rose less dramatically in the medical group (27.0%, 30.7%, and 36.0%, respectively) [8]. Old age (> 65 years) has been associated with increased community-acquired pneumonia mortality [23] and ARDS mortality (OR per additional 10 year, 1.27; 95% CI, 1.07-1.50) [24]. In the current study, the ICU mortality was similar in the three age groups, but the hospital mortality was significantly higher in patients aged ≥ 80 years having a higher adjusted mortality risk compared to younger age groups (50–64.9 and 65–79.9 years). This was observed in different admission types, except for admissions due to neurologic disease and hospital-acquired pneumonia. This could be because patients aged ≥ 80 years had high prevalence of chronic illnesses and functional disability in our study.

The findings of this study should be interpreted in the light of its strengths and limitations. Strengths include the large sample size. Limitations include being a monocenter study and lack of data on post-ICU care processes, which may have affected hospital outcome, and on long-term outcomes such as cognitive function and disability. Critical illness in old people has been associated with decline in cognitive function. Analysis of data from a prospective cohort study of 2929 individuals ≥ 65 year-old without dementia showed that adjusted hazard ratio for incident dementia was 1.4 following a noncritical illness hospitalization (95% CI, 1.1-1.7; p = 0.001) and 2.3 following a critical illness hospitalization (95% CI, 0.9-5.7; p = 0.09) [25]. This may be one of the reasons for increased mortality after ICU discharge.

## Conclusions

In conclusion, we have found that patients aged ≥ 80 years accounted for a significant proportion of patients admitted to a tertiary-care ICU in Saudi Arabia. However, their proportion was lower than those of developed countries likely due to the younger Saudi population. The treating intensivists supported them with life sustaining interventions, such as mechanical ventilation and renal replacement therapy, similar to younger groups, but the Do-Not-Resuscitate practice was more common in them. More than half of them died in the hospital with age ≥ 80 years being an independent risk factor for hospital mortality.

### Key messages

- Patients aged ≥ 80 years represented a significant portion of ICU admissions (7.9% of all admissions and 12.8% of patients aged ≥ 50 years).
- They received life support measures such as mechanical ventilation and renal replacement therapy similar to younger patients. However, they had higher frequency of Do-Not-Resuscitate orders.
- Patients aged > 80 years had higher hospital mortality than the younger patients mostly after ICU discharge.
- Age ≥ 80 years was associated with almost 2 times increase in the adjusted hospital mortality risk compared with age 50-64.9 years.

## Abbreviations

APACHE: Acute Physiology and Chronic Health Evaluation
CI: Confidence interval
ICU: Intensive care unit
INR: International Normalized Ratio
MPM: Mortality Probability Model
OR: Odds ratio.
